# Iron Deficiency Anemia in Female Adolescents: An Epidemiological Study From Eastern Iran—A Cross‐Sectional Study

**DOI:** 10.1002/hsr2.72878

**Published:** 2026-09-07

**Authors:** Mohammad Javad Yousefi, Sepideh Sahebi, Freshteh Osmani, Mobina Seghatoleslami, Elnaz Abbasi, Mohammad Hossein Soltani, Fatemeh Mezginejad

**Affiliations:** ^1^ Student Research Committee Birjand University of Medical Sciences Birjand Iran; ^2^ Department of Epidemiology and Biostatistics, Faculty of Health Torbat Heydarieh University of Medical Sciences Torbat Heydarieh Iran; ^3^ Department of Hematology, School of Allied Medicine, Cardiovascular Diseases Research Center Birjand University of Medical Sciences Birjand Iran

**Keywords:** anemia, high school students, IDA, iron deficiency

## Abstract

**Background and Aims:**

Iron deficiency is the most widespread nutritional deficiency worldwide. Although iron deficiency can affect women throughout their reproductive years, late adolescence (16–18 years) represents a particularly vulnerable period because of rapid growth, increased iron requirements, establishment of menstrual cycles, and dietary transitions. Identifying iron deficiency during this critical developmental stage may facilitate early intervention before entry into adulthood and the broader reproductive period. Since there is no confirmed information on the prevalence of iron deficiency in this high‐risk age group in South Khorasan Province, this study aimed to assess the prevalence of IDA among female high school students in Birjand.

**Methods:**

This study was conducted on blood samples from participants to analyze serum ferritin, total iron‐binding capacity (TIBC), iron as well as complete blood count (CBC). Written consent was obtained before sample collection. The samples were then sent to a medical diagnostic facility for analysis. IDA was diagnosed based on the criteria established by the World Health Organization (WHO).

**Results:**

The study included 148 participants, with an average age of 17.47 ± 1.209 years (range: 16–18). Among the 143 participants, 15.5% were diagnosed with anemia, while 7.4% were classified as iron deficiency anemia. The mean levels of ferritin, TIBC, and serum iron were 44.199 ng/mL, 327.79 µg/dL, and 97.31 µg/dL, respectively. Out of all, 8.8% of individuals were assigned to the first phase of progress to IDA, whereas 2.7% were assigned to the second phase.

**Conclusion:**

Findings of the present study showed that iron deficiency is an increasing concern in this population and needs to be addressed.

## 1 Background

Anemia is a significant global public health concern, affecting over 1.62 billion people and more than 25% of the world's population [1]. Iron deficiency is the most widespread micronutrient deficiency worldwide and remains a major public health issue, particularly in developing countries. According to the WHO, the majority of the global population is iron‐deficient, with at least one‐third experiencing anemia due to insufficient iron levels [2].

IDA occurs when the body's iron stores are depleted, resulting in insufficient iron for the normal synthesis of hemoglobin. This condition is considered a significant economic concern, as it reduces work productivity and adversely affects children's growth, development, and educational attainment [3]. On average, individuals who do not menstruate lose approximately 1 mg of iron per day. In contrast, menstruating women experience iron losses ranging from 6 mg to over 10 mg per menstrual cycle, with total losses potentially reaching up to 42 mg per cycle, depending on the severity of bleeding [4].

The prevalence of anemia, iron deficiency (ID), and iron deficiency anemia (IDA) varies considerably across populations and is influenced by cultural, socioeconomic, demographic, and geographic factors [5, 6, 7]. Anemia and IDA are particularly common in low‐ and middle‐income countries and disproportionately affect women of reproductive age and children [6, 8, 9]. In Asia and Africa, the burden of anemia and iron deficiency remains especially high among these vulnerable groups [8]. In the United States, the prevalence of anemia has been reported as 8.5% among young women and 9.5% among adolescent females aged 13–19 years [10]. Among Saudi female students, the prevalence of anemia ranges from 20% to 39.9% [11], while a cross‐sectional study in Iraq reported anemia in 56% of women [12]. In Iran, the prevalence of anemia and IDA among high school girls has been reported as 9.5% and 4.5%, respectively, in Semnan, and 13.5% and 9.3%, respectively, in Yazd [13, 14]. Additionally, anemia has been reported in 49.8% of schoolchildren in Kazakhstan [15].

The pathogenesis of IDA in adults is often attributed to the depletion of iron reserves due to persistent blood loss, although periods of rapid growth, such as the first year of life and adolescence, also necessitate iron supplementation. In premenopausal women, menstrual blood loss is considered the primary cause of iron deficiency, while in males and postmenopausal women, intestinal bleeding is the most common cause of IDA [9]. Other contributing factors include esophageal varices, gastric resection, gastrointestinal disorders, diarrhea, and, in some cases, intense physical activity, which may lead to impaired iron absorption [4, 16].

Fatigue and reduced quality of life are the primary consequences of anemia and IDA significantly affects the mental development of children and adolescents. Importantly, iron deficiency may adversely affect health even before anemia develops. Non‐anemic iron deficiency has been associated with fatigue, impaired cognitive performance, reduced attention and memory, decreased physical capacity, poorer academic achievement, and diminished quality of life. Therefore, early identification of iron deficiency is important not only to prevent anemia but also to reduce the broader functional consequences of iron depletion. There are also evidences suggesting that IDA can impair vision and hearing [4]. Individuals with IDA may experience symptoms such as palpitations, shortness of breath, pallor, weakness, decreased physical endurance, mood swings, reduced concentration, and learning difficulties. Furthermore, even with proper treatment, iron deficiency and IDA can lead to irreversible developmental and growth impairments, as well as neurological deficits, especially in infants [4].

Several countries have already implemented screening programs to detect iron deficiency and the resulting anemia in at‐risk populations at an early stage [17]. Due to the high prevalence of this nutritional deficiency in certain nations, iron supplementation is commonly added to frequently consumed foods to help address the issue [12, 17, 18].

Late adolescent girls (15–19 years) represent a particularly vulnerable population for iron deficiency and IDA because of the combined effects of rapid growth, increased physiological iron requirements, the establishment of regular menstrual cycles, and dietary transitions [19, 20]. Iron deficiency during this critical developmental period may adversely affect physical performance, cognitive function, educational achievement, and future reproductive health [19]. Despite the public health importance of this condition, data regarding the prevalence of anemia and IDA among adolescent girls in South Khorasan Province remain limited.

South Khorasan Province, located in eastern Iran, is characterized by a predominantly arid climate, dispersed rural populations, and socioeconomic challenges that may influence dietary patterns and nutritional status. Limited access to diverse food sources, potential reliance on plant‐based diets with lower iron bioavailability, and regional health disparities may increase vulnerability to iron deficiency among adolescent girls. Therefore, generating region‐specific evidence is essential to support targeted public health interventions and nutritional policies. Accordingly, the present study aimed to determine the prevalence of anemia and IDA among high school girls in Birjand, South Khorasan Province, Iran.

## 2 Methods

### 2.1 Study Design and Participants

This study conducted on 16–18 years old females during September to December 2024. The inclusion criteria were: (1) female sex, (2) age between 16 and 18 years, (3) enrollment in a high school in Birjand, and (4) willingness to participate in the study. Students who had not yet started menstruating at the time of the study were excluded from the sample. Students who had not yet started menstruating at the time of the study were excluded from the sample. Participants were recruited through a convenience sampling, and the final sample size was established based on the availability of eligible cases during the study period. Written informed consent was obtained from parents or legal guardians of participants younger than 18 years, while assent was obtained from the adolescents themselves. Participants aged 18 years or older provided written informed consent independently.

### 2.2 Data Collection and Process

After obtaining written consent from the parents, blood samples were taken from each participant (in one tube containing EDTA anticoagulant for CBC and another without anticoagulant for serum testing) and sent to a medical diagnostic laboratory for analysis. Laboratory results were provided to both the participants and their parents. Students with abnormal findings were advised to seek medical evaluation. In the second phase, students with hemoglobin levels below 12 g/dL, as defined by the WHO as anemic, were further tested for serum iron, TIBC, and ferritin. According to WHO criteria, IDA is diagnosed if ferritin levels are below 15 ng/L, transferrin saturation is below 16%, serum iron is below 50 μg/dL, or TIBC levels exceed 400 μg/L. Based on laboratory findings, participants were classified into three phases according to predefined objective criteria.

Phase 1 (Depleted iron stores): serum ferritin < 15 ng/mL, with normal hemoglobin levels (≥ 12 g/dL).

Phase 2 (Iron deficiency without anemia): serum ferritin < 15 ng/mL and/or serum iron < 50 μg/dL, with hemoglobin ≥ 12 g/dL.

Phase 3 (IDA): hemoglobin < 12 g/dL in combination with serum ferritin < 15 ng/mL.

### 2.3 Data Analysis

The data were described using descriptive statistics, including measures of central tendency and dispersion indices (mean, standard deviation, and frequency (%)). Continuous variables were assessed for normality using the Shapiro–Wilk test. Comparisons between two groups were performed using Student's *t*‐test as appropriate. Comparisons among more than two groups were conducted using one‐way ANOVA test followed by post hoc analyses with Bonferroni correction. Categorical variables were analyzed using the *χ* ^2^ test. Effect sizes were calculated for all primary comparisons. All statistical tests were two‐sided unless otherwise specified, with a priori significance level set at *α* = 0.05. *p* value of less than 0.05 was considered statistically significant.

## 3 Results

The mean age of the 148 students who participated in this study was 17.47 ± 1.209 years. Ten participants (6.8%) had a hematocrit level below 35%, while 14 subjects (9.5%) had hemoglobin levels below 12 g/dL. Of the 148 individuals, 15.5% (23 participants) were diagnosed with anemia, which 7.4% of them were classified as IDA. The mean ferritin, TIBC, and serum iron levels among the participants were 44.199 ng/mL, 327.79 µg/dL, and 97.31 µg/dL, respectively. Table 1 presents the CBC results of the study participants, expressed as mean ± standard deviation.

**Table 1:** CBC parameters of the study participants.

Baso (×10^3^/µL)	Eos (×10^3^/µL)	Nut (×10^3^/µL)	Mono (×10^3^/µL)	Lym (×10^3^/µL)	RDW (%)	Plt (10^3^/µL)	MCHC (g/dL)	MCH (pg)	MCV (fL)	Hct (%)	Hb (g/dL)	RBC (10^6^/µL)	WBC (10^3^/µL)
0.02 ± 0.02	0.17 ± 0.12	4.34 ± 3.42	0.55 ± 0.23	3.12 ± 2.69	14.27 ± 10.23	299.49 ± 64.90	33.59 ± 1.02	28.40 ± 2.88	84.43 ± 7.36	40.33 ± 3.29	13.56 ± 1.23	4.79 ± 0.41	8.03 ± 7.73

*Note:* Data are presented as mean ± standard deviation (SD) (148 students).

IDA affected 7.4% (11) of the 148 students surveyed. Table 2 presents the ferritin, TIBC, mean serum iron levels, and age categorized by diagnosis.

**Table 2:** Mean serum iron, ferritin, TIBC, and age levels by phases.

Number	Age	Ferritin	TIBC	Fe	Parameters
					Phases
13	16.85 ± 0.98	13.02 ± 3.8	352.35 ± 88.12	92.15 ± 34.15	Phase 1
4	17.00 ± 1.15	9.97 ± 4.49	407.00 ± 23.98	48.50 ± 13.17	Phase 2
11	17.18 ± 1.25	9.54 ± 7.34	365.00 ± 116.46	41.82 ± 21.97	Phase 3
120	17.58 ± 1.23	51.88 ± 43.81	374.57 ± 329.96	100.15 ± 40.10	Normal

*Note:* Phase 1 (depleted iron stores): serum ferritin < 15 ng/mL, with normal hemoglobin levels (≥ 12 g/dL). Phase 2 (iron deficiency without anemia): serum ferritin < 15 ng/mL and/or serum iron < 50 μg/dL, with hemoglobin ≥ 12 g/dL. Phase 3 (iron deficiency anemia, IDA): hemoglobin < 12 g/dL in combination with serum ferritin < 15 ng/mL.

Among the 120 participants classified as non‐anemic, 8 (6.7%) had serum ferritin levels between 15 and 20 ng/mL, indicating reduced iron stores and a potential risk of developing iron deficiency. No significant association was observed between age and iron status (*p* > 0.05). Based on iron status classification, 13 participants (8.8%) were categorized as Phase 1 (depleted iron stores), and 4 participants (2.7%) were categorized as Phase 2 (iron deficiency without anemia). IDA (Phase 3) was identified in 11 participants (7.4%). Due to the cross‐sectional design of the study, progression between these phases could not be assessed.

## 4 Discussion

The findings of the current study indicate that 15.5% of the students were anemic, which is consistent with another research in this field. For instance, studies have reported that 13.5% of high school girls in Yazd suffer from anemia [14], and 15.3% of Indian girls between the ages of 5 and 15 are affected by anemia [2]. However, a study of female medical students in Tehran found a lower prevalence of 3.8%, which contrasts with the results of our study [21]. These discrepancies may be attributed to differences in the population characteristics and sample size. This discrepancy may be partially explained by socioeconomic and healthcare‐related challenges that exist in some areas of South Khorasan Province, which may influence access to health services, nutritional status, and health‐related behaviors. Other potential explanations for this discrepancy include limited public health literacy and lower engagement in health‐promoting behaviors.

The findings of this study also contrast with other research that reports significantly higher rates of anemia. A study of Canadian females aged 14–17 revealed an anemia prevalence of 43% [22]. In a study conducted in Nagpur, 91.1% of females aged 10–19 were found to be anemic [23]. Additionally, 49.8% of schoolchildren in Kazakhstan were reported to have anemia [15]. The study by Akramipour and colleagues, which investigated the prevalence of anemia among high school females aged 14–20 in Kermanshah, reported a prevalence of 21.4% [24]. Similarly, Abedini and Aghamolaei's study on the prevalence of anemia and IDA in high school females in Bandar Abbas found an anemia prevalence of 20.5% [18]. This variation may be attributed to differences in the populations studied. The present study focused on high school students; a group particularly susceptible to anemia due to their age‐related physiological demands. Additionally, regional factors—such as dietary iron intake, the prioritization of health and medical care, and the extent of screening programs—may contribute to this disparity. Methodological differences across studies could also account for the observed variations.

In this study, 7.4% of participants were found to have IDA. The prevalence of IDA in other studies varies, with 4.5% reported among high school girls in Semnan, 2.12% in Kermanshah, and 9.3% in Yazd [13, 14, 24]. Similar to our findings, an 8.3% prevalence was observed among female medical students in Tehran [13, 14, 21]. However, our results differ from several other studies. For instance, IDA was found to affect 44.8% of high school girls in Jolfa and 43.7% of those in Varamin. Geographical differences may contribute to these variations in prevalence. International studies report a prevalence of 14% in Canada, 21.8% in Indonesia, 32.4% in Kazakhstan, and 88.2% in Turkey [15, 22, 25, 26, 27]. Factors such as differences in research periods, iron supplementation practices, variations in the age range of participants, the criteria used to define anemia based on hemoglobin levels, or the socioeconomic background of the study populations may also account for these discrepancies. The variation in reported prevalence of iron deficiency across different studies may partly reflect the use of varying diagnostic thresholds, particularly for serum ferritin. While the World Health Organization (WHO) defines iron deficiency in apparently healthy individuals as ferritin levels < 15 µg/L, some organizations and clinical guidelines recommend higher cut‐offs (e.g., 30 or even 50 µg/L), especially in settings with inflammation or chronic disease [28]. Such methodological differences can substantially influence prevalence estimates and should be considered when comparing epidemiological findings across regions. Although anemia was observed in 15.5% of participants, only 7.4% met the criteria for IDA. This finding suggests that factors other than iron deficiency may contribute to anemia in this population. Potential explanations include deficiencies of vitamin B12 or folate, inherited hemoglobin disorders, chronic inflammatory conditions, or other nutritional and non‐nutritional causes. As these factors were not assessed in the present study, further investigations are warranted to clarify the etiology of anemia among adolescents.

However, IDA remains a global health issue, its severity varies significantly, likely due to factors such as dietary habits and environmental influences that affect the intake of iron absorption enhancers and inhibitors in different study populations. Socioeconomic status, as well as proper food preparation and consumption, may also play significant roles in influencing iron levels. Given the serious health implications of IDA, especially in underdeveloped nations, it is crucial to ensure that diets are balanced and provide adequate energy, protein, and essential nutrients, including iron. Public health efforts should focus on educating the community about strategies to enhance iron absorption through diet in order to effectively address this widespread issue. The findings of this study can inform screening efforts and support evidence‐based health‐related decision‐making. Furthermore, public education initiatives highlighting the importance of anemia and its treatment options are feasible and recommended. Where necessary, iron supplementation may be considered as part of a comprehensive preventive strategy. Previous studies and WHO recommendations have demonstrated that iron supplementation programs among adolescent girls can improve iron stores and reduce the risk of iron deficiency and IDA, particularly in populations with a high burden of nutritional deficiencies [29]. While iron supplementation and educational programs may help mitigate iron deficiency, it should be considered that multiple environmental, dietary, and health‐related factors can contribute to iron status among adolescents. Therefore, targeted interventions should consider these broader determinants, and further studies are warranted to explore their contribution to iron deficiency in this population.

## 5 Conclusions

The findings of our study indicate a modest prevalence of IDA among adolescent females in Birjand. Adolescent females are at increased risk for iron deficiency, and further research and targeted preventive interventions are needed to better address this issue and improve adolescent health outcomes in this population.

## Author Contributions

**Mohammad Javad Yousefi:** conceptualization, methodology, data curation, writing – original draft. **Sepideh Sahebi:** conceptualization, methodology, data curation. **Freshteh Osmani:** conceptualization, methodology, formal analysis. **Mobina Seghatoleslami:** conceptualization, data curation, methodology. **Elnaz Abbasi:** conceptualization, methodology, data curation. **Mohammad Hossein Soltani:** conceptualization, methodology, formal analysis. **Fatemeh Mezginejad:** conceptualization, methodology, writing – review and editing. All individuals contributed to the ideation and design of this study. All authors agree to the manuscript, and this article has not been published elsewhere. All authors have read and approved the final version of the manuscript.

## Disclosure

Fatemeh Mezginejad affirms that this manuscript is an honest, accurate, and transparent account of the study being reported; that no important aspects of the study have been omitted; and that any discrepancies from the study as planned (and, if relevant, registered) have been explained.

## Ethics Statement

This study was approved and supported by the Ethics Committee of Birjand University of Medical Sciences.

## Consent

Informed consent was obtained from all participants prior to their inclusion in the study.

## Conflicts of Interest

The authors declare no conflicts of interest.

## Data Availability

The data that support the findings of this study are available on request from the corresponding author. The data are not publicly available due to privacy or ethical restrictions. Fatemeh Mezginejad had full access to all of the data in this study and takes complete responsibility for the integrity of the data and the accuracy of the data analysis.
